# Color Phenomena in Polymer Fracture*

**DOI:** 10.6028/jres.067A.062

**Published:** 1963-12-01

**Authors:** Sanford B. Newman, Irvin Wolock

## Abstract

Thin layers derived from the matrix are produced in the fracture plane of some polymers prior to ultimate failure. A certain amount of the evidence would indicate that these layers consist of an oriented array of molecules. Fracture isolates these molecular segments in a thin film with physical properties differing from that of the matrix in which they originated. These films produce interference phenomena which in some cases are useful in elucidating structure and origin.

## 1. Introduction

It is difficult to ascertain when the strong colors on the fracture surfaces of polymers were first observed. In 1956 Busse, Orowan, and Neimark [1]^1^ exhibited some surfaces produced by fracturing large notched plates of polymethyl methacrylate. About a year before, somewhat more diffuse colors had been observed on tensile specimens broken under static conditions [2] at the National Bureau of Standards [3]. Although Wolock et al. [4] and Berry [5] both reported observing colors associated with specific fracture markings, the first detailed discussion of the phenomenon was published by Higuchi [6]. Many of the same observations were made independently and reported by Wolock et al. [7] and Berry [8].

It is entirely possible, of course, that colored fracture surfaces were observed earlier than the period mentioned. Higuchi, for example, indicates that the experiments of Benbow and Roesler [9] must have produced such surfaces. Thus far almost all colored surfaces that have been studied have been produced on polymethyl methacrylate.

## 2. Fracture Color Patterns

The colors arising from interference in subsurface cracks are not considered in this discussion. Although they produce an optical effect which often appears quite close to the surface its origin is obvious and easily ascertained.

There is considerable variety in the color patterns obtained by various fracturing techniques. Those found on statically tested tensile specimens are weak, usually located about the periphery of the mirror area and are found on relatively few specimens. They are not usually associated with an individual structure, such as parabolas, but cover an area which encompasses a number of figures or structures without any specificity of color or tint for each element. Fractures exhibited by Busse and his coworkers were more brilliant and formed bands transverse to the direction of fracture. Higuchi used notched sheets 3–5 nun thick and 40 mm wide. The exact conditions of test are not described. His figures show a variety of patterns. Most of the conic sections are differentiated in color from their surround and occasional transverse bands cause a reversal of colors in the banded area. He finds that the colors on the two fracture surfaces are generally complementary and that the red areas are raised and the green are depressed relative to the specimen matrix.

**Figure 1:**
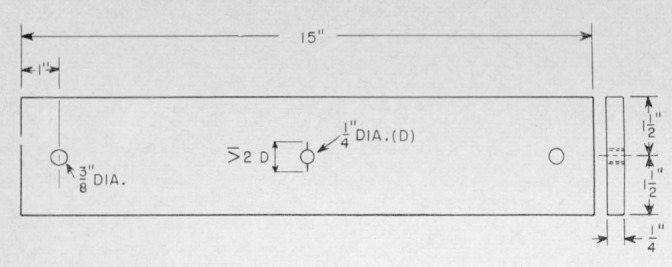
Schematic diagram of crack propagation specimen. Small variations in overall dimensions and position of loading pin holes do not significantly affect fracture characteristics.

Wolock [7] produced similar colors on fracture surfaces of polymethyl methacrylate specimens which had been broken by rapid overstress. Films of diffuse color were formed on surfaces of cast polystyrene broken in the same fashion. Specific colors, however, were not related to the individual structures on the polystyrene fracture faces. Somewhat later, Wolock noted the intense differential color produced on the relatively smooth fracture surfaces of specimens of polymethyl methacrylate tested for resistance to crack propagation. Many of the observations to be discussed were made on fractures produced in these specimens (fig. 1), which were broken by loading through a cross head moving at 0.008 mm/sec (0.02 in./min). These contain four types of color patterns or characteristics (fig. 9). The first is observed in the slow-growth area and actually consists of two types of patterns, namely, multicolored areas (fig. 9b) and single-colored areas (fig. 9a). The next pattern (fig. 9c) is composed of irregularly shaped elongated figures. These figures develop, in the third pattern of this series (fig. 9d) into oriented forms that lead, in many cases, into parabolas. The last area (fig. 9e) is darker (less reflective) with little or no color and containing many crowded parabolas in which only the foci are very distinct. The significance of the geometric markings on the fracture surfaces have been dealt with extensively in the literature [10–12] and will not be discussed in this paper.

A characteristic blue color is also found on fracture surfaces of hot-stretched polymethyl methacrylate. These surfaces are produced by cleaving the stretched sheets through the thickness dimension. The colors are not associated with specific markings.

## 3. Optics of Color Formation

It can be demonstrated that the strongly colored areas of the fracture surfaces produced in crack propagation specimens are covered with a soft thin film. The presence of this film is a prerequisite for the development of color. In figure 10 a soft swab has been wiped across a fracture surface with removal of essentially all of the tint. Under room conditions the color will disappear from crack propagation specimen surfaces over a period of months. The time for color disappearance varies greatly, however. Colors on ordinary tensile specimens and on cleaved, stretched material faded in the course of a few weeks, whereas those of Busse et al. [1] were still intense after almost a year. Storage at temperatures of 50 to 60 °C causes rapid loss of color in crack propagation specimens in well under an hour. Loss of color under these circumstances is not necessarily associated with complete loss of the film. Even when the colors have completely disappeared an oily film still occupies the surface. The disappearance of color by aging or heating takes place by fading of the original tint without any pronounced color shift [6, 7].

Concentrated vapors of a number of organic solvents such as toluene and methyl chloride will often eliminate colors in a matter of minutes. In this case a change in tint is involved as can be seen in figure 11. The first color changes seem concentrated along level difference lines and a few other structures on the fracture face.

Another method of affecting color intensity of parts of the film is by exposure to low pressure. This can be demonstrated by placing one of the matching surfaces of a crack propagation specimen in a container maintained at approximately 1·10^−5^ mm Hg pressure. After removal it will be found that almost all of the color is removed from the slow growth area. The color intensity over the remainder of the fracture is apparently the same as that of the matching surface which had been stored outside of the vacuum chamber. Microscopic examination and micromanipulation of the film on the slow-growth area of the fracture surface indicates that this portion is thicker than the rest of the film.

**Figure 2:**
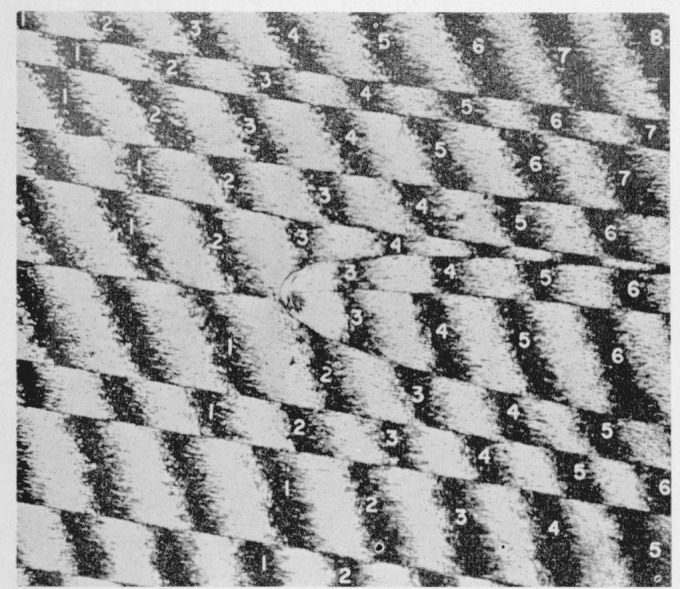
Multiple-beam interferogram of parabola-bearing portion of a polymethyl methacrylate crack propagation specimen fracture surface. Numbers identify components of the same fringe. Source was the 5461 Å mercury line.

The fact that much of the surface is little affected by high vacuum makes it possible to apply the methods of multiple-beam interference microscopy to the study of the color phenomenon. Figure 2 is a multiple-beam interferogram of a portion of a fracture produced in a crack propagation specimen. The field shows a portion of the first parabola-containing area. A layer of silver has been deposited over the surface film and the fringe displacement over the composite surface is shown. It would appear that the adjacent areas covered by the contribution of different secondary fractures are consistently displaced by about 1500 Å. When the color is removed by aging or heating before the specimen is prepared for interference microscopy this displacement remains about the same.

**Figure 3:**
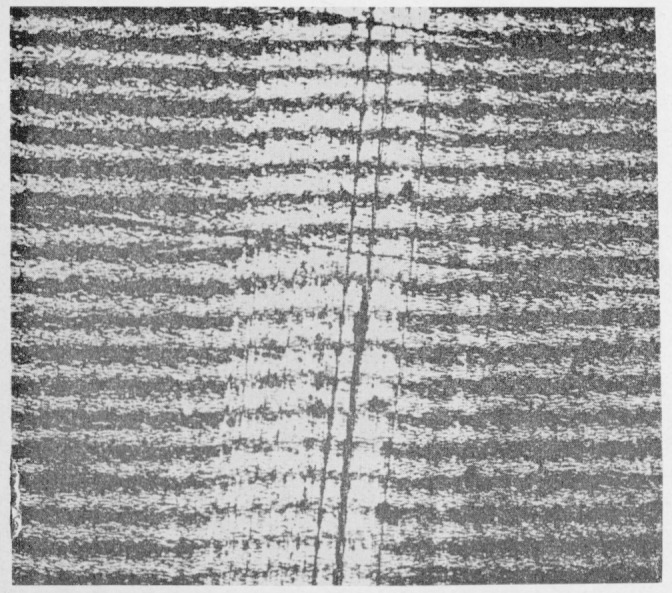
Multiple-beam interferogram of the swabbed area of the fracture surface of a crack propagation specimen of polymethyl methacrylate. Source was the 5461 Å mercury line.

Assuming first-order interference Higuchi [6] has estimated a film thickness range of 400 to 700 m*μ* from the observed colors. Using a soft swab to remove the film produces a swath with a depth of less than 200 m*μ* in the surface as measured by multiple-beam techniques (fig. 3). This experiment was repeated by focusing the radiation from a zirconium arc lamp on a small area of the fracture surface until all the color had disappeared and continuing the treatment until the film was scarcely detectable in the irradiated portion by microscopical examination. The depth of the circular depression was again found to be somewhat less than 200 m*μ*. Although the film in the slow growth area may exceed this figure, its sensitivity to vacuum prevents direct measurement. Figure 3 also shows the increased reflectivity of the treated area. Apparently swabbing or heating tends to produce a surface that is smoother than the original face of the film.

**Figure 4:**
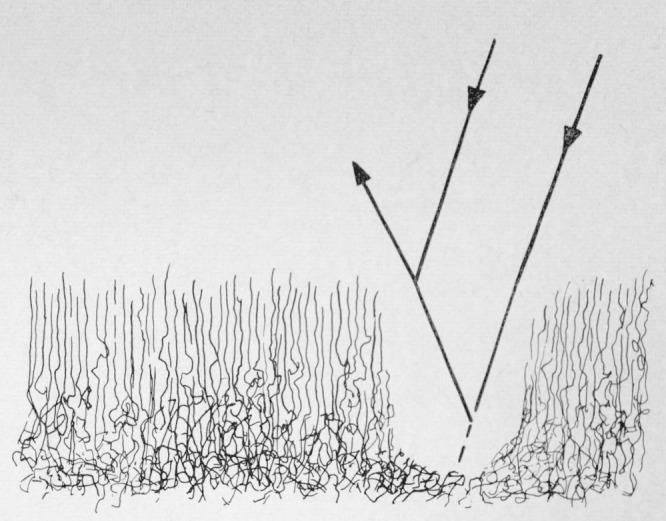
Diagrammatic representation of interference in the oriented film of the fracture of polymethyl methacraylate. (Modified from Higuchi [6])

The presence of the film led Higuchi [6], Wolock et al. [7], and Berry [5, 8] to postulate its formation by the pulling out of chain ends and the scission of chains. These segments would have been extended in the fracture process and thus could have a somewhat different density than the matrix. The chain segments in only a relatively small thickness of material would be extended during the fracture process. Oriented layer and matrix would differ sufficiently in refractive index to lend themselves to the development of thin film interference as shown figure 4. In any given region, an average height would predominate which would result in the color observed. As the chain segments composing the film relax, the distribution of heights remains the same but the number of segments remaining in the film decreases so that the same color is present but is diminished in intensity. This would account for the fading observed with no change in color. Solvent vapors produce different changes in the structure reflected by the change in color withdrawal. The derivation of the film from the matrix would lead to a structure whose properties would differ from the matrix to the greater degree at the exposed face and tend to blend into the matrix at the other limit. Although the blending section close to the matrix could contribute to the optical effect, it would be difficult to establish its boundary by the methods described. Hence failure to realize the theoretical film thickness is probably to be expected.

Tolansky’s thin film technique [13] permits corroboration of this explanation of the origin of the color phenomenon with a simple model. A fracture surface with the colors removed by aging or some other method is covered with a totally reflected film of silver *in vacuo.* A drop of a dilute solution of Canada balsam in xylene is placed on the silvered surface and allowed to drain by tilting the specimen. The surface of the film of Canada balsam in contact with the fracture surface replicates the topography of the surface. The upper surface of the film, however, does not contour the fracture surface but stretches itself into a single plane for considerable distances. On illuminating such a surface two-beam interference fringes will be formed. Figure 12 illustrates a portion of a fracture colored by thin film interference. The wrinkling probably caused by solvent attack on the silver and plastic substrate is noticeable but otherwise there is considerable similarity to the fracture-produced colors. Even the slight coloring observed in the rough, low-reflecting zone is almost identical (fig. 13) with that observed on the original fracture surface.

**Figure 5:**
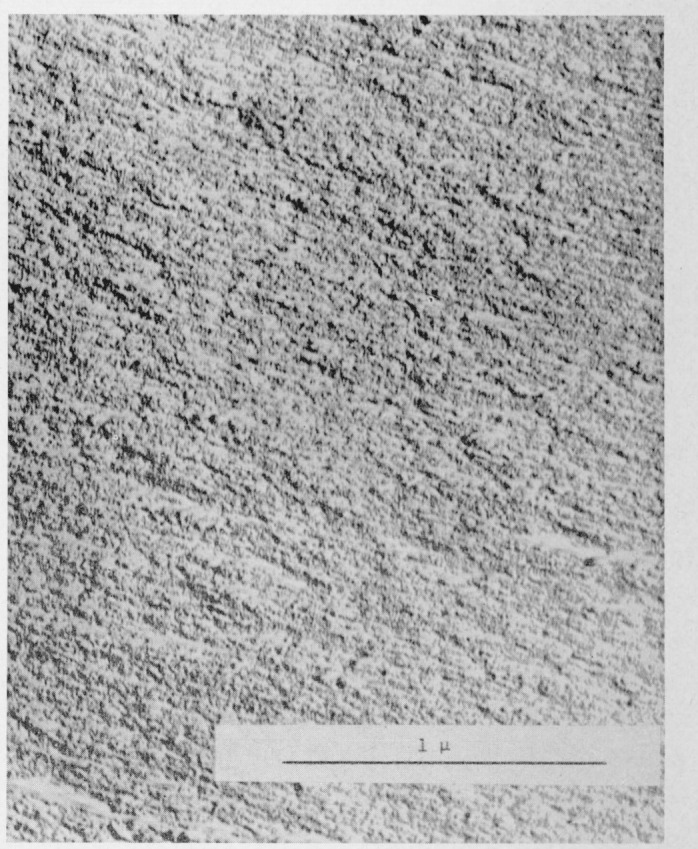
Electron micrograph of a replica of the fracture surface of a crack propagation specimen of polymethly methacrylate. The area shown is from the slow-growth region.

**Figure 6:**
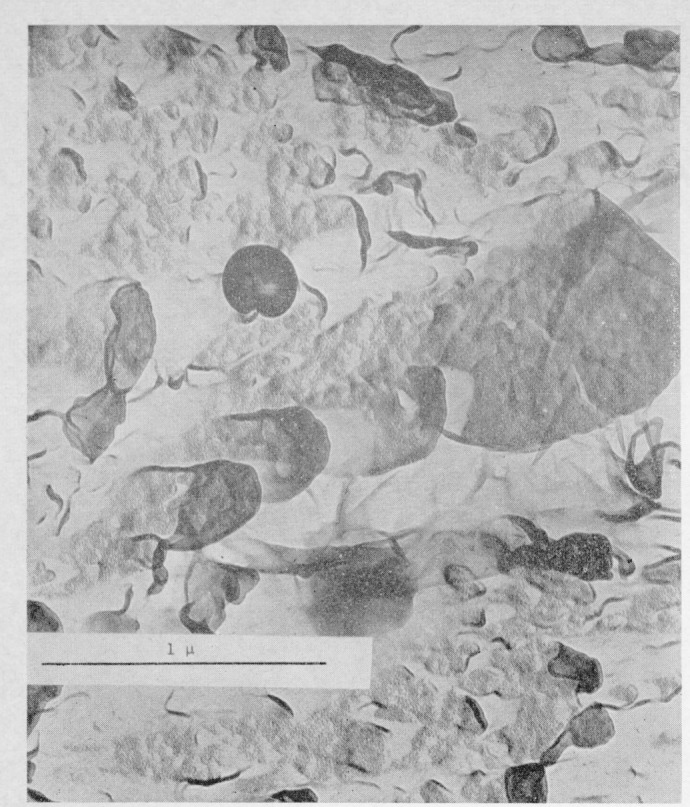
Electron micrograph of a replica of the fracture surface of a crack propagation specimen of polymethyl methacrylate. The area shown is from the crowded parabola region.

Considerable fringe broadening is apparent in the interferogram in figure 2. This is at a minimum in the initial, smooth, slow growth area and increases toward the crowded parabola portion. An electron micrograph of these two areas, figures 5 and 6, shows the change in surface granularity responsible for the fringe broadening. Although the surface is sufficiently smooth up to the first parabolas to permit the use of multiple-beam interference, the final surface is so rough that the method fails. Figure 6 reveals this surface to be sculptured with thin leaf-like projections. It is doubtful that a fragile film in the millimicron thickness range could be formed there. Only isolated fragments of color are found in this area.

Replicas for electron microscopy were produced by allowing a dilute solution of methyl cellulose to dry on the fracture surface. Despite its limited resolution, which is well illustrated in figure 6, this method was chosen because it would impose the least abuse on the polymer matrix. On stripping the film from a surface originally bearing color, the surface was found to be colorless. A carbon replica was made from the negative and shadowed by vacuum deposition. The loss of color would be caused either by the stripping of all or part of the interference film with the cellulosic replica or by disorienting it with the replicating solution.

**Figure 7:**
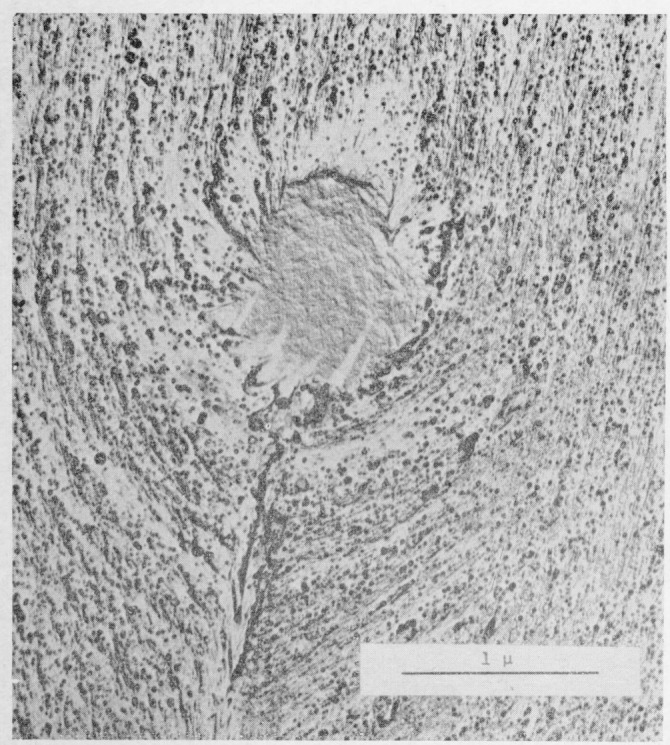
Electron micrograph of a replica of a fracture surface of polymethyl methacrylate broken by rapid overstress. The surface is covered by droplets derived from the surface film.

Before replicating the surfaces in figures 5 and 6, an attempt was made to remove thoroughly the surface film by heating for 96 h at 50°C. For purposes of comparison replicas were also prepared from a surface bearing bright interference colors. In most cases the replicas from the unheated colored surfaces bore numerous droplets over a considerable portion of the fields examined, as in figure 7. It would seem that a portion of the film is removed and subsequently disrupted by the replicating process. These observations, moreover, pinpoint one of the difficulties encountered in preparing high resolution replicas of a polymer fracture.

This discussion has been limited almost entirely to polymethyl methacrylate. Attempts to produce color on a number of other polymers by varying loading rates and thermal conditions have been largely unsuccessful. However, polystyrene has occasionally yielded colored surfaces, as shown in figure 14.

## 4. Color Patterns in Crazing

Niegisch [14] has recently convincingly demonstrated the presence of oriented material in craze cracks produced in long-time tests of polycarbonates. Although this polymer did not exhibit brittle fracture under the test conditions, the ultimate elongation being about 100 percent, some evidence of similar “craze matter’’ in polystyrene and polymethyl methacrylate was also presented. From these data, Spurr and Niegisch [15] have offered an attractive theory for the development and structure of craze cracks in thermoplastics. Probably the most important contribution of their observations is the support they provide for the conclusion that the appearance of crazing does not mean the end of structural integrity or continuity in the stressed specimen.

**Figure 8:**
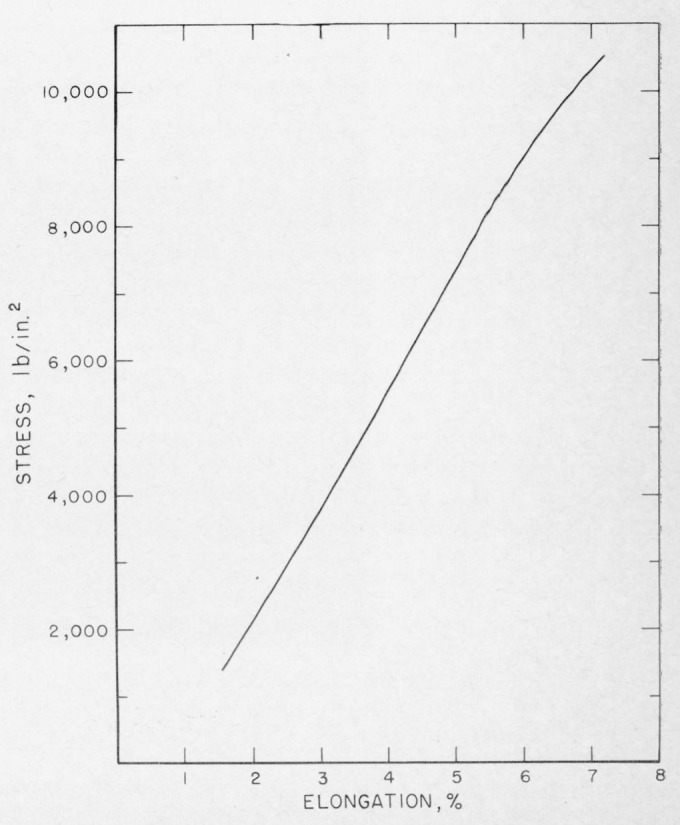
Stress-elongation characteristics of acrylonitrile-styrene copolymer.

**Figure 10:**
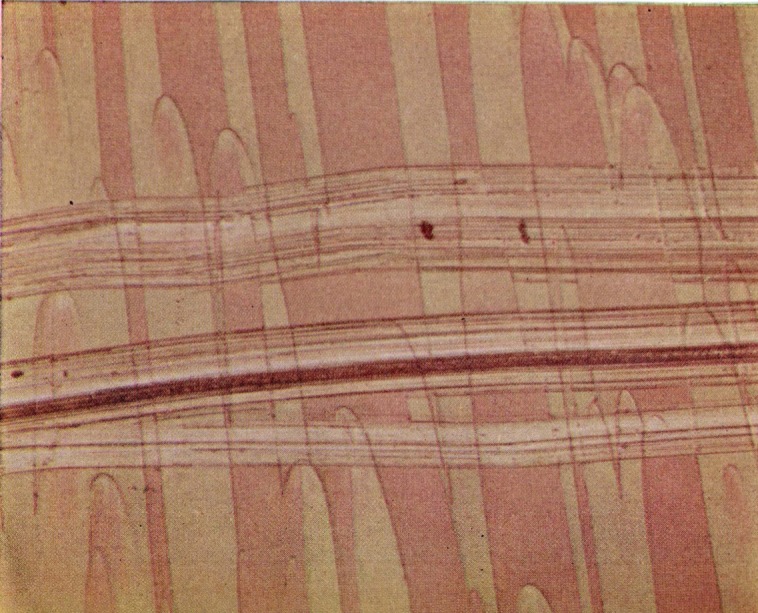
Area of the parabola-bearing region on the surface of a fracture produced in a crack propagation specimen of poly methyl methacrylate. A soft swab has produced swaths through the surface film. Note the loss of color in the swaths which have not disturbed the geometric structures.

**Figure 11:**
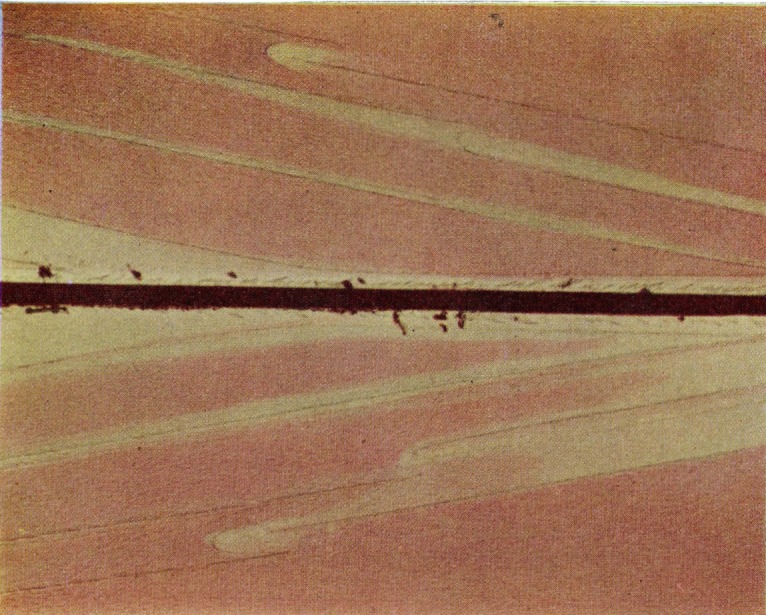
Part of the surface of a fracture produced in a crack propagation specimen of polymethyl methacrylate. The surface shows the effect of exposure to vapors of methyl chloride (upper half). The lower section is the untreated matching surface.

**Figure 12:**
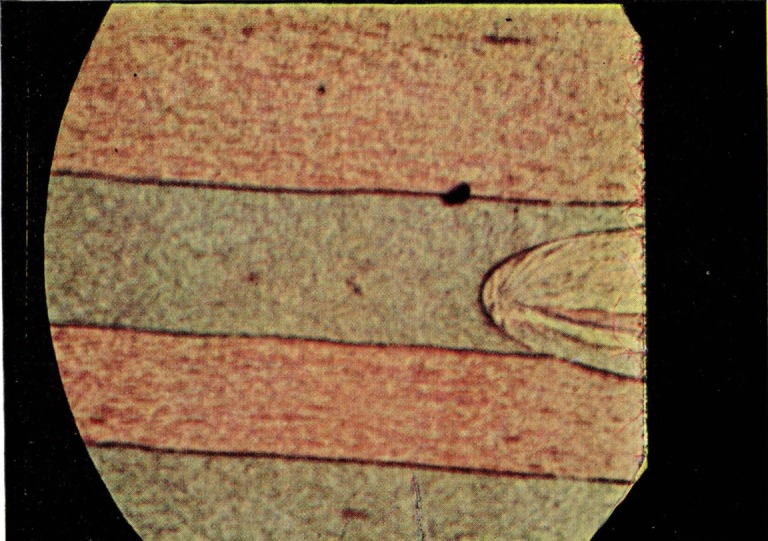
Fracture surface colors produced by thin-film interference. A reflecting layer of silver was deposited on the fracture surface *in vacuo* and subsequently a thin solution of Canada balsam was deposited over the silver.

**Figure 13:**
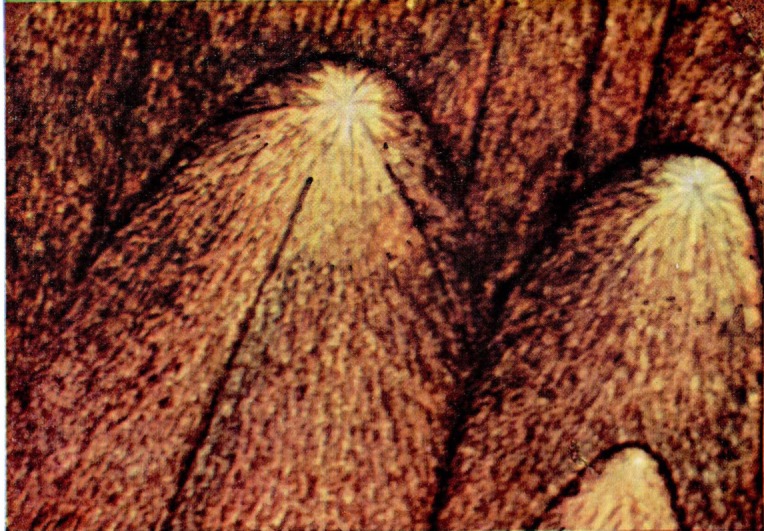
Thin-film interference on the crowded parabola portion of the surface of a fracture produced in a crack propagation specimen of polymethyl methacrylate.

**Figure 14:**
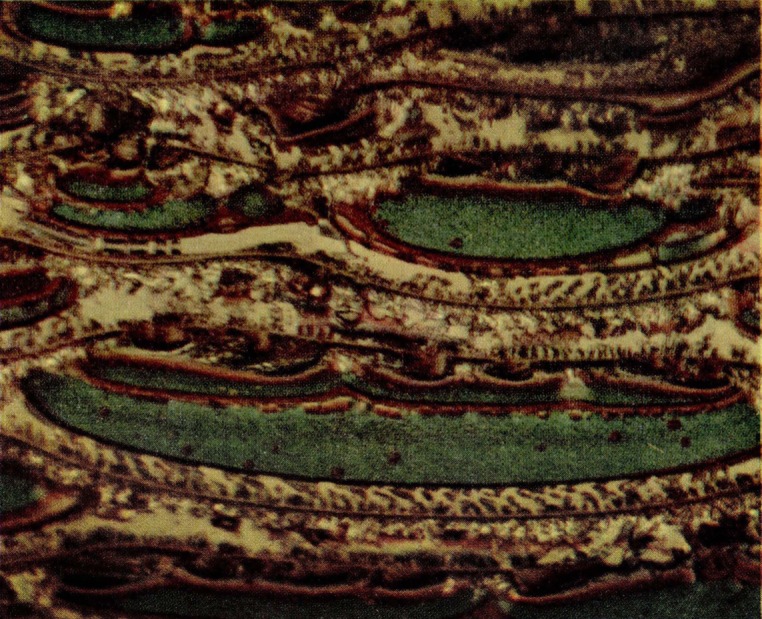
Fracture surface of a tensile specimen of molded polystyrene.

**Figure 15:**
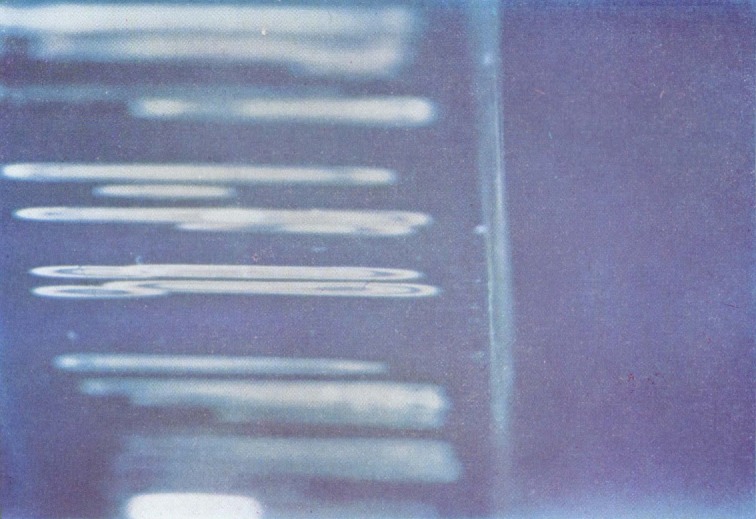
Large internal craze cracks in a tensile specimen of molded acrylonitrile-styrene copolymer. The specimen was loaded to failure through a crosshead moving at 0.02 in./min.

The matter in polycarbonate cracks is visible by difference in refractive index probably coupled with some scattering from micropores. However, during some routine tests of standard tensile specimens [2] of acrylonitrile-styrene copolymer, a striking color phenomenon was also noted in the craze cracks of this material, as seen in figure 15. These specimens were injection molded and the stressed skin was apparently responsible for the formation of internal cracks which sometimes encompassed one-half or more of the specimen cross section. These tensile specimens, when loaded at a head speed of 0.008 mm/sec have the load-elongation characteristics shown in figure 8. The large internal cracks suddenly appear apparently full grown at the top of the straight line portion of the curve but without any discontinuities in loading. Ultimate failure occurs through one of these large cracks.

**Figure 16:**
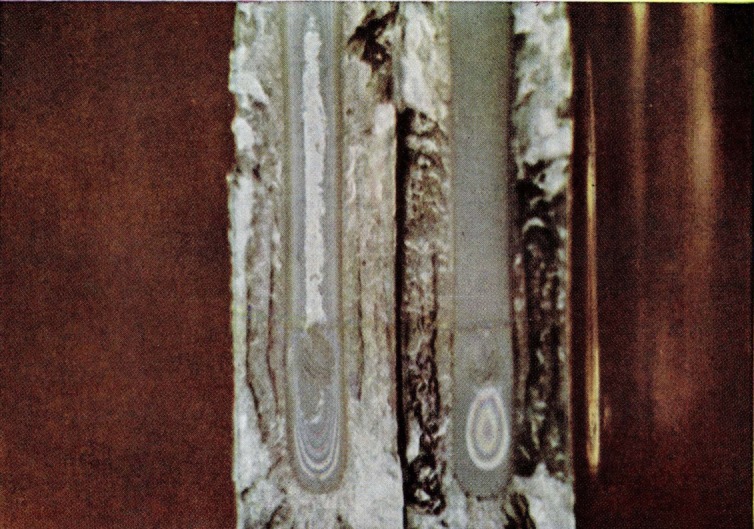
Matching fracture faces of a tensile specimen of acrylonitrile-styrene copolymer. The specimen was loaded to failure through a crosshead moving at 0.02 in./min.

It was originally assumed that the optical effect was due to an interference system involving a void. On examining the fracture surface, however, it was found that a pattern similar to that noted in the intact craze cracks was found on one surface or parts of one surface, as shown in figure 16. The matching surface was devoid of color. Colors observed in the intact cracks are more intense than those found on fracture surfaces. Critical microscopical examination established the presence of a film on the color-bearing surface. Unlike the film found on polymethyl methacrylate fracture surfaces, the film is integral and shears cleanly from one of the crack faces. Edges of the film are sometimes curled free of the matrix on which they lie but mechanical removal of any significant amount of film appears impractical.

**Figure 17:**
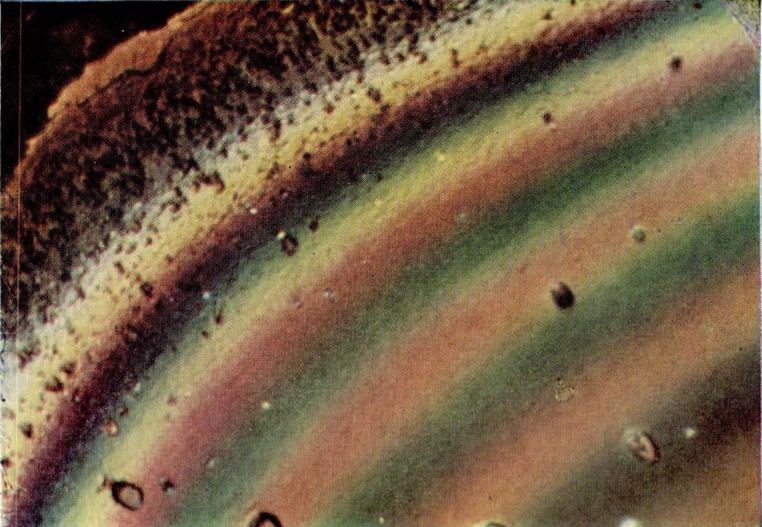
Detail from a fractured craze crack of acrylonitrile-styrene copolymer. The periphery of the crack is in the upper left-hand corner. The film displays fringes of at least four orders.

If the film-bearing surface of the fracture is examined at higher magnification, some additional details can be resolved. As in the intact cracks the peripheral area of the fractured craze is covered with fringes of equal thickness. The film in figure 17 bears first to fourth order colors which indicates a thickness of approximately 2 *μ*. Thicknesses of this magnitude are not uncommon although many cracks with much thinner films are found. In general, however, the film is much thicker than those found on the fracture surfaces discussed in the earlier section.

**Figure 18:**
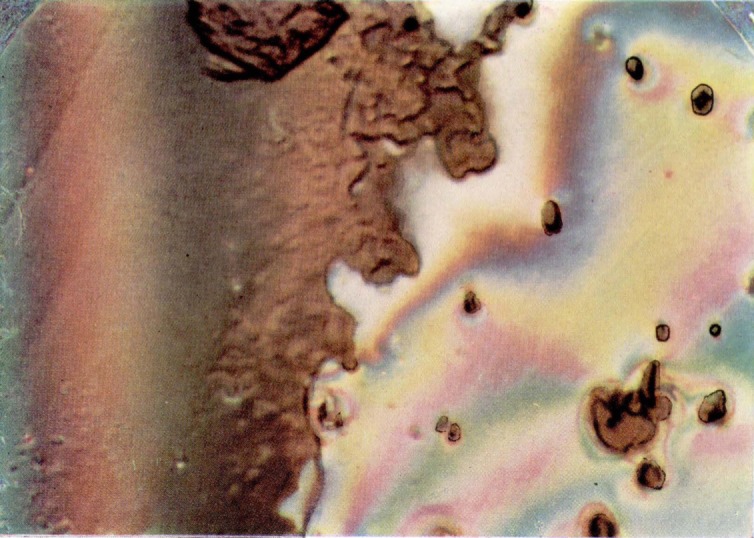
Detail from a fractured craze on acrylonitrile-styrene copolymer. The area bearing fringes of equal thickness is at the left. The bright fringes cover a portion of the film which is slightly wrinkled and also stretched in the vicinity of the holes.

The center of the film-bearing area is likely to show more disorder. Colors are bright and the fringe pattern has contours that seem unrelated to film thickness. Such an area occupies about half the field in figure 18. The film is wrinkled and stretched, especially in the regions surrounding the small holes. Evidently it has been slightly dislodged from the substrate during fracturing.

**Figure 19:**
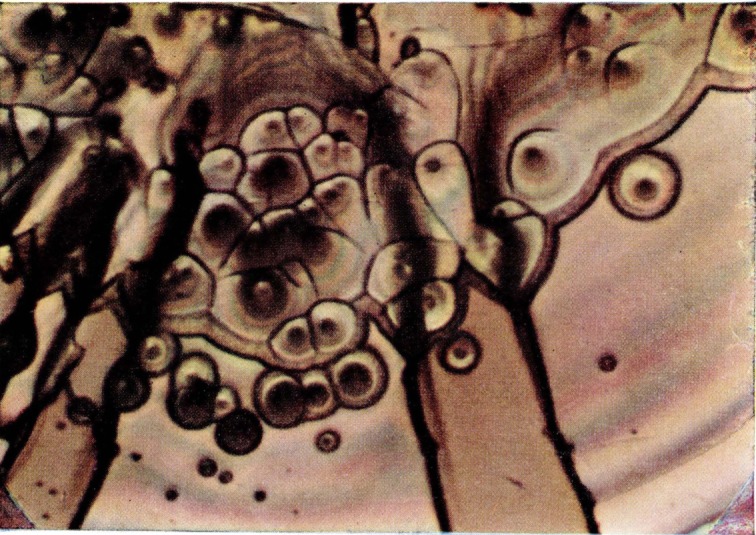
Detail from the center of a fractured craze crack of acrylonitrile-styrene copolymer. Conic sections characteristic of brittle failure are associated with the surface film.

In a few small areas the conic sections characteristic of brittle failure sculpture the face of the fractured craze crack as shown in figure 19. If the crack surfaces are connected by a thin layer of oriented molecules as originally suggested by Bessonov and Kuvshinskii [16] and developed by Spurr and Niegisch [15], the connection may not be as tenuous as might be suggested by a consideration of the properties of the film on polymethyl methacrylate surfaces. Besides its association with the brittle mode of failure, the crack material is much more resistant to heat, aging, and solvents. Exposure in an oven at 70 °C for 48 h produced little change in the appearance of the film on the acrylonitrile-styrene copolymer fracture surface. Prolonged heating at this temperature caused some fading of the colors and a great increase in the pitting, which is visible in the periphery of the film in figure 17. Colors in the intact cracks were unaffected by weeks of heating at 70 °C or exposure to solvent vapors that did not cause gross matrix deterioration.

Spurr and Niegisch [15] detected “craze matter” in cracks of polystyrene and polymethyl methacrylate. Its presence was much more difficult to demonstrate than in polycarbonate and it was possible to record photographically. Recently, however, the description of the work of Bessonov and Kuvshinskii [17] became available. They had observed phenomena closely related to those described in our acrylonitrile-styrene copolymer in specimens of “annealed polystyrene.” Their specimens with a section of 2×2 mm and a free length of 15 mm were maintained at a temperature of 110 °C for 30 min and then cooled rapidly by plunging them into liquid nitrogen. These specimens were then loaded to failure at a rate of deformation equal to 0.04 mm/sec. The pretreatment resulted in the formation of a stressed skin and a 20 percent increase in tensile strength. Failure was preceded by the formation of large internal cracks as in the acrylonitrile-stvrene copolymer. They report that the material filling these cracks remains on the fracture surface. Part of this forms interference fringes and clings to the matrix but part can be stripped. They suggested that the film consists of the bundles of linear polymers discussed by Hsiao and Sauer [18] and Kargin et al. [19].

Since certain details necessary for comparison with previous results were not given in this report, the work was repeated using standard tensile specimens [2]. The preliminary heating was extended to 2 hr in view of the larger cross section. These specimens were loaded to failure at a head speed of 0.008 mm/sec Large internal cracks formed shortly before failure and failure occurred through cracks encompassing one-third to one-half of the cross section. No color phenomenon was visible in any of the cracks on gross examination.

On microscopically examining the fracture surface a few faint interference bands could be observed. The film responsible for these adhered strongly to one of the surfaces. The remainder of the crack was partly covered on both faces by a transparent film. The film was partly detached and partly loosely attached to the surface. It was easily stripped and pieces 5 or 6 mm^2^ were removed with forceps. The film was lower in refractive index than the matrix. It appears to have good physical properties and the relatively large areas available should permit the use of transmission electron microscopy, infrared spectroscopy, and other instrumental techniques in elucidating their structure and mode of formation.

Attempts to produce large cracks in specimens of polymethyl methacrylate by this technique have thus far been unsuccessful.

## 5. Summary

Thin layers derived from the matrix are produced in the fracture plane of some polymers prior to ultimate failure. A certain amount of the evidence would indicate that these layers consist of an oriented array of molecules. Fracture isolates these molecular segments in a thin film with physical properties differing from that of the matrix in which they originated. These films produce interference phenomena which in some cases are useful in elucidating structure and origin.

The suggestion has been made that these oriented layers provide structural continuity in polymers after optical inhomogeneities are produced by stress. This is supported, at least superficially, by observations on polycarbonate and acrylonitrile-styrene copolymer. In these materials intact craze cracks appear to be filled with homogeneous or oriented material. The film observed in intact cracks of “annealed” polystryene, however, shows some disorder. Folds and wrinkles are almost always present and it is difficult to attribute any load-bearing function to this structure.

It is possible that the two types of film formation may not be as closely related as has been assumed during much of this discussion. Further experimentation may provide the necessary data for supporting the assumption. If the mechanism proposed for the formation of these films is correct, we would expect to detect them on a wide range of polymers when failure is produced under suitable conditions. The search for, and study of, these films is continuing.

## Figures and Tables

**Figure 9a:**
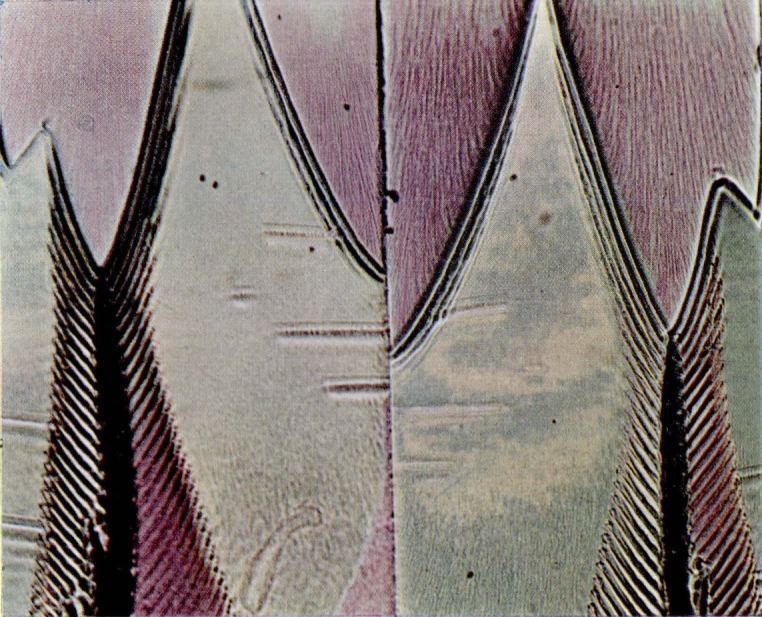
Single colored area in the slow-growth region of a fracture produced in a crack propagation specimen of polymethyl methacrylate. The matching faces are shown side by side.

**Figure 9b:**
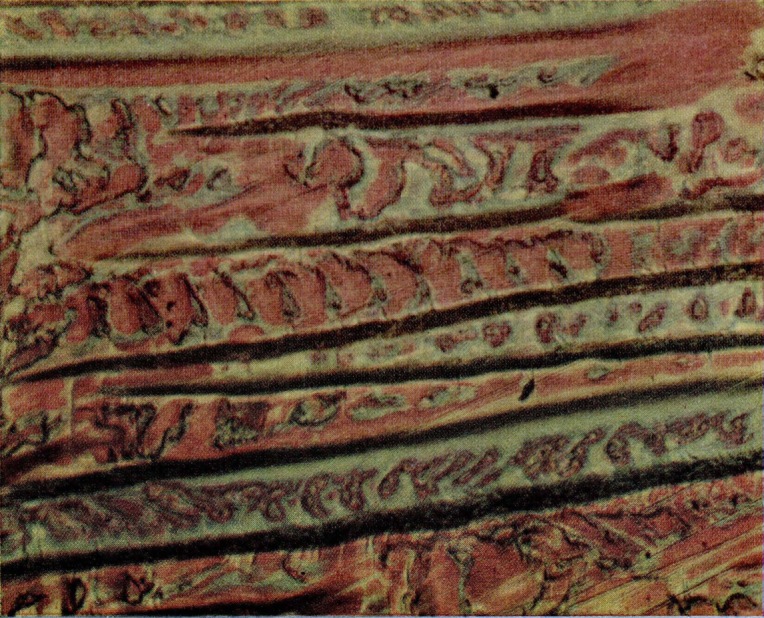
Multicolored area in the slow-growth region of a fracture produced in a crack propagation specimen of polymethyl methacrylate.

**Figure 9c:**
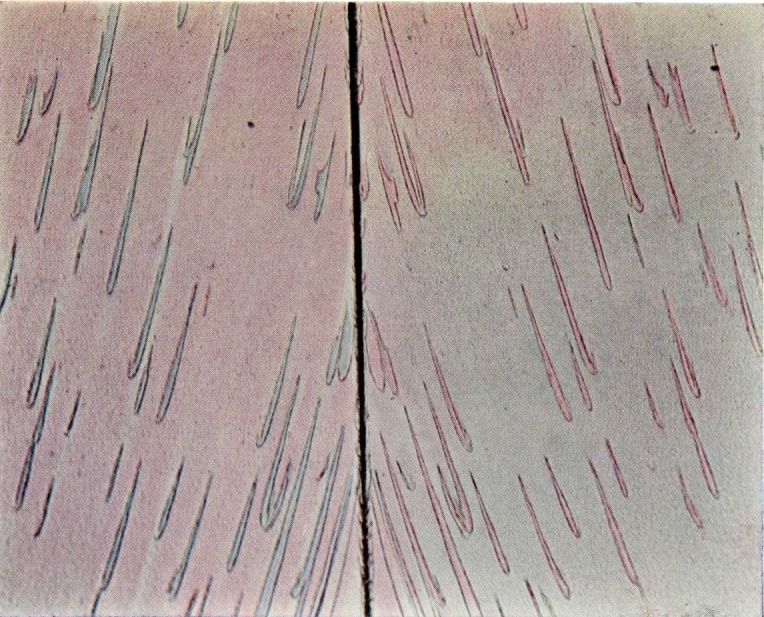
Area of irregularly shaped elongated figures on a fracture surface produced in a crack propagation specimen of polymethyl methacrylate. The matching faces are shown side by side.

**Figure 9d:**
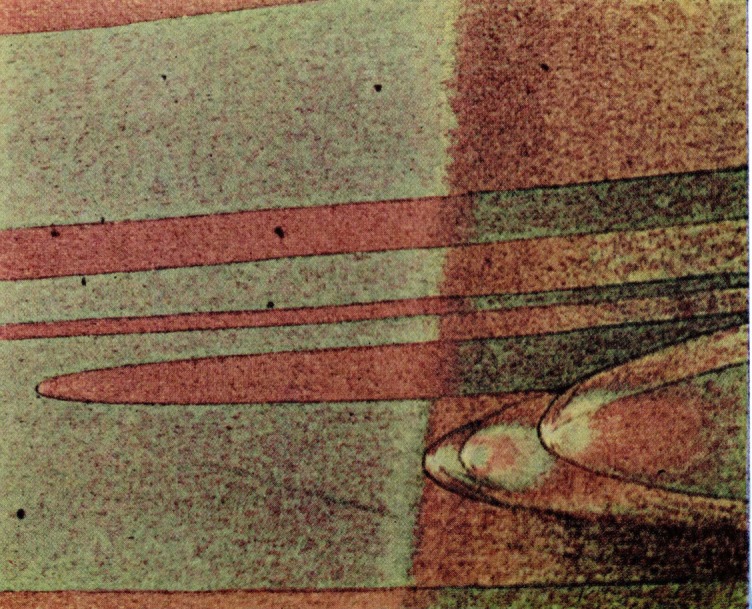
Detail from an area on the fracture face of a crack propagation specimen of polymethyl methacrylate in which the elongated figures merge into the parabola-containing region. Note the same structures bearing complementary colors on opposite sides of the transverse color interface.

**Figure 9e:**
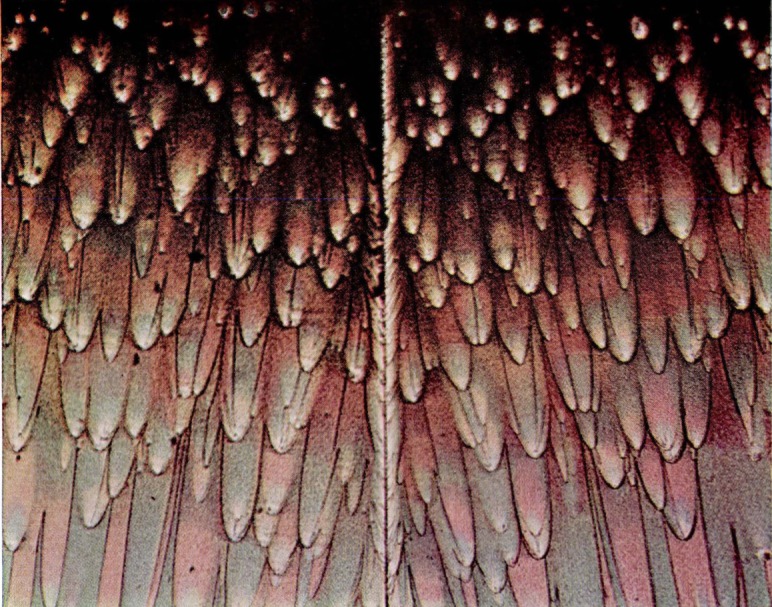
Area of crowded parabolas on a fracture surface produced in a crack propagation specimen of polymethyl methacrylate. The matching faces arc side by side.

## Footnotes

1 Figures in brackets indicate the literature references at the end of this paper
